# Oral healthcare seeking behavior of Malaysian adults in urban and rural areas: findings from the National Health and Morbidity Survey 2019

**DOI:** 10.1186/s12903-023-03470-5

**Published:** 2023-10-05

**Authors:** Yeung R’ong Tan, Suhana Jawahir, Jennifer Geraldine Doss

**Affiliations:** 1 Department of Community Oral Health & Clinical Prevention, Faculty of Dentistry, Universiti Malaya, 50603 Wilayah Persekutuan Kuala Lumpur, Malaysia; 2 Institute for Health Systems Research, National Institutes of Health, Ministry of Health Malaysia, Blok B2, Kompleks NIH, No. 1, Jalan Setia Murni U13/52, Seksyen U13 Setia Alam, 40170 Shah Alam, Selangor Malaysia

**Keywords:** Oral healthcare seeking behavior, Urban population, Rural population, Adults, Malaysia

## Abstract

**Background:**

The development and implementation of appropriate strategies to enhance oral health in the community can be aided by an understanding of oral healthcare seeking behavior among urban and rural populations. The purpose of this study was to identify the factors associated with oral healthcare seeking behavior of the Malaysians in urban and rural locations who self-reported dental problems.

**Methods:**

The National Health and Morbidity Survey 2019, a cross-sectional nationwide household survey that focused on non-institutionalised Malaysians, provided the data for this study on adults in Malaysia who were 18 years of age and older. A two-stage stratified random sampling technique was employed to ensure national representativeness. Data was collected using a multilingual (Malay and English), structured, and validated questionnaire via face-to-face interviews from July to October 2019. The dependent variable was oral healthcare seeking behavior (sought oral healthcare and self-medication). Independent variables were predisposing, enabling and health needs factor based on Andersen’s Behavioral Model. Descriptive statistics were used to describe the characteristics and oral healthcare seeking behavior of the respondents. The relationship between the independent and dependent variables were investigated using multivariable logistic regression analysis.

**Results:**

The analysis comprised a total of 10,134 respondents, representing about 18.2 million Malaysian adults aged 18 and above. The overall prevalence of Malaysian adults who self-reported dental problems was low (5.5%) and was slightly higher in the rural than urban population. Almost half sought treatment from healthcare practitioners, and almost a quarter self-medicated. Ethnicity was associated with seeking healthcare and self-medication among urban dwellers. Among the rural population, income level was associated with seeking healthcare while education level was associated with self-medication.

**Conclusion:**

Disparities in oral healthcare seeking behaviors exist between Malaysians living in urban and rural areas. Future policies should adopt focused strategies that concentrate on oral healthcare accessibility and health literacy of the vulnerable and rural populations to achieve the best oral healthcare for this population group.

## Background

One of the key factors affecting the oral health of a community is their oral healthcare seeking behavior (OHSB). It determines how oral health services are used, thus the general population's oral health [1]. Any action taken by individuals who believe they are experiencing a health issue or are ill in order to find a suitable treatment is referred to as health or care-seeking behaviour [1].

Numerous studies [2–4] have discussed the variables that significantly influence people's healthcare seeking behavior during disease episodes, as well as how often they use formal or informal oral healthcare facilities and self-care, or home remedies. These studies have demonstrated that a number of variables, including socioeconomic status, sex, age, the severity and type of illness, access to services, and perceived service quality, can affect a person's decision to seek care through a particular process or from a healthcare practitioner (HCP) [3–5]. In addition, marital status, educational attainment, occupation, and health beliefs all affect decisions regarding utilizing oral healthcare in Malaysia [6]. However, information on healthcare seeking behavior of Malaysian adults in urban and rural areas pertaining to oral health issues is currently not available.

Self-medication or use of medication without getting advice from a HCP has been linked to antibiotic misuse which may lead to drug-related complications and the emergence of antibiotic resistance [7]. In addition, it may mask disease severity leading to misdiagnosis, which predisposes further complications of disease, increases cost, and delays initiation of appropriate dental treatment [8]. Even though Malaysia's community pharmacies are growing, and more individuals may have access to over-the-counter medications, the Ministry of Health (MOH) has put restrictions on prescription-only medications including antibiotics [9]. According to other studies, a variety of factors contribute to antibiotic resistance, including antibiotic abuse and overuse [10], and primarily because of the non-prescription distribution of antibiotics [11] despite the fact that the national guidelines on antibiotics are easily accessible to the general public and healthcare professionals [12].

Geographic factors influence access to healthcare considerably [13, 14] which in turn contribute to the gap in health equity across the urban and rural setting [15]. Access to dental care is important for the population to receive basic preventive services and education, which would enable early intervention of oral diseases [16]. This is a challenge in rural areas where resources are limited and where the population experiences poorer health status, awareness, attitudes, and greater knowledge gaps (poorer health literacy) than those from better-off communities [17–19]. Studies have also indicated a lower prevalence of the population who sought dental care from HCPs in rural areas than in urban areas of Malaysia when they encountered oral health problems [20–22]. The Malaysian healthcare system is built around a geographically extensive healthcare delivery system designed to provide everyone with access to public health services, including those in rural and urban areas [14]. In Malaysia, a gazetted area is classified as an urban area if its combined population is 10,000 or more, and as a rural region if it is less than 10,000 [23]. Due to geographic constraints [24], among other things, Malaysia's structured public healthcare system and equitable healthcare financing do not guarantee equal access to healthcare [14].

Malaysia has a dichotomous healthcare system consisting of a public sector that is heavily subsidised through general taxation, and a fee-for-service private sector [14]. The World Health Organization recognises Malaysia to have achieved Universal Health Coverage since the 1990s [25]. The public sector is accessible to all Malaysian citizens and permanent residents at a nominal fee, with fee exemptions for vulnerable population such as the poor, disabled, and elderly [14]. A comprehensive network of dental care facilities located at health clinics, standalone clinics, hospitals, schools and institutions are provided by the public sector [26]. Dental care facilities are also available in the Urban Transformation Centres (UTCs) and Rural Transformation Centres (RTCs). The UTCs provided daily outpatient dental services while the RTCs provided outreach dental services. Delivery of outreach dental services is also established through Mobile Dental Clinics (buses, trailers, lorries and caravans) and mobile dental teams to the schoolchildren and populations in sub-urban and remote areas. Non-governmental organisations also provide targeted community dental services for the vulnerable population [27, 28]. Overall, the Malaysian health system is designed to provide dental care access to the entire population across the urban and rural settings [14]. Despite the various efforts by the Ministry of Health Malaysia to improve the oral health of its population, inequality in oral health utilization persists very noticeably [6].

The disparities in oral healthcare utilization among Malaysia’s adult population could be reduced by better understanding the factors that affect the behaviour of people seeking oral healthcare within urban and rural areas. The only available information on the prevalence of OHSB among Malaysians is among adults with type II diabetes [29] and from technical reports that summarize findings from previous iterations of the National Health and Morbidity Survey (NHMS) [21, 22, 30]. The NHMS technical reports offer an overview of prevalence rates for the overall population of Malaysia which encompassed individuals aged 13 years and above while this study focused specifically on adults aged 18 years and older, aligning with the legal age of majority in Malaysia. Information about OHSB and prevalence among Malaysian adults with dental problems in urban and rural areas is scarce. Unlike our current investigation, the earlier studies did not delve into a detailed analysis of differences in OHSB between urban and rural contexts. There is also no published study on factors associated with healthcare seeking and self-medication among Malaysian adults who self-reported dental problems in urban and rural areas that can be generalized to the entire Malaysian population. Several studies have used healthcare seeking and self-medication as proxies for healthcare seeking behaviors [13, 31, 32]. In this study, the Andersen behavioural model was adopted due to its popularity and ease of application in understanding healthcare utilisation [33]. The model suggests that predisposing (tendency to use the services), enabling (resources required to utilise healthcare), and health need (perceived need for healthcare) factors influence OHSB [33].

Understanding OHSB and its associated factors in urban and rural areas is important to reduce dental care inequalities that occur across different geographic localities and population groups. Thus, in this study, we set out to: (1) identify the prevalence of self-reported dental problems among Malaysian adults according to location; (2) identify the OHSB of Malaysian adults who self-reported dental problems, according to location; and (3) identify the factors related to the OHSB of Malaysian adults who self-reported dental problems, according to location.

## Methods

### Study design and sampling

Using a subgroup of persons aged 18 years and above from the National Health and Morbidity Survey (NHMS) 2019, secondary data analysis was carried out.

### Data source

Data were obtained from the National Health and Morbidity Survey (NHMS) 2019, a cross-sectional national household survey that targeted all Malaysians who were not institutionalised. The NHMS 2019 provides nationally representative estimates of non-communicable diseases, healthcare demand, and health literacy in Malaysia. Complex sampling is a sampling method that takes into account the different characteristics of the population being sampled considering that the study was a nationwide population survey. To ensure nationwide inclusivity in this study, a two-stage stratified random sampling technique was employed [20, 34]. This means that the population was divided into strata or groups, and then a random sample was selected from each stratum. This method involved categorizing the sample into two distinct strata: the Primary stratum, encompassing all 13 states and 3 federal territories in Malaysia, and the Secondary stratum, comprising urban and rural sub-strata within the Primary stratum. The sampling process comprised two stages: firstly, the selection of Primary Sampling Units (PSUs), which were Enumeration Blocks (EBs), followed by the selection of Secondary Sampling Units (SSUs), which were Living Quarters (LQs) within the chosen EBs. The Department of Statistics Malaysia (DOSM) [23] used the population density of gazetted regions to classify EBs in Malaysia as urban or rural in the NHMS 2019. An EB with a combined population of 10,000 or higher is considered an urban area, while an EB with a combined population of less than 10,000 is considered a rural area. The complex sampling design was used because it is more likely to produce a representative sample of the population than simple random sampling. This is because the design takes into account the different characteristics of the population, which helps to ensure that all groups are represented in the sample. Data collection took place between July and October 2019, overseen by trained data enumerators. The collection involved face-to-face interviews utilizing a bilingual (Malay and English), structured, and validated questionnaire. This questionnaire was incorporated into an application and deployed on tablets as the primary data collection tool. The data amassed were securely stored and backed up using Secure Digital (SD) cards. After undergoing a quality check, the data were subsequently transferred to a central system.

In the survey, residents meeting the criteria from chosen households were invited to participate. For residences found vacant or closed during the initial visit, follow-up visits were conducted at least thrice to ensure the desired sample size was attained. Detailed information about NHMS 2019 is available in its official report [20, 34]. The Declaration of Helsinki's principles were upheld as the study was conducted. Prior to the interviews, all participants provided their written, informed consent. The National Health and Morbidity Survey 2019 was authorised by the Medical Research and Ethics Committee (MREC), Ministry of Health Malaysia (NMRR-18–3085-44207).

### Data analysis

The analysis only considered respondents who provided complete data on all variables.

#### Oral healthcare seeking behaviors (Dependent variables)

In the present study, two dependent variables from the data source were included namely: (1) sought healthcare, and (2) self-medication. It is important to note that this pertains exclusively to oral health issues and does not include other health problems. Those who self-reported dental problems in the last two weeks before the interview were respondents who answered “yes” to the question “In the last 2 weeks, did you experience any dental problems such as toothache or sensitive tooth, swollen gums with or without pus discharge, loss of teeth, denture problems, mouth ulcers, or jaw pain?”.

Their OHSB (yes or no) were based on whether they sought treatment/medication or advice from HCPs (sought healthcare) or took medicine without advice from HCPs (self-medication) in the last two weeks prior to data collection. This was assessed among those who self-reported dental problems. The phrase "sought healthcare" in this study was defined as "seek treatment/medication or guidance from HCPs". HCPs included traditional and alternative medicine practitioners (e.g., Islamic, Chinese, and Ayurvedic medicine practitioners) as well as contemporary HCPs like community pharmacists. Self-medication was defined as "taking medicine without consulting a healthcare professional" The OHSBs were then assessed in relation to the potential determinants (independent variables).

#### Potential determinants (Independent variables)

##### Predisposing factors

In the present study, the predisposing factors included sociodemographic characteristics including sex, ethnicity, age, education level, and marital status. Based on the age distribution pattern, the respondents' age in years was divided into three categories: "18–34", "35–59", and "60 + years". No formal education refers to respondents who have never attended school or who have not completed primary school, whereas ‘primary’ education level refers to those who have completed Standard Six. Individuals with ‘Secondary’ education level were those with at least five years of secondary school. ‘Tertiary’ education level refers to those who have completed Form Six or received academic certificates, diplomas, or degrees.

##### Enabling factors

The enabling factors included employment status (government, private, self-employed, or unemployed); total monthly household income which was grouped into quintiles (quintile 1 (Q1) (RM0 – RM1,100), quintile 2 (Q2) (RM1,108 – RM2,100), quintile 3 (Q3) (RM2,103 – RM3,400), quintile 4 (Q4) (RM3,410 – RM5,900), or quintile 5 (Q5) (RM5,930 – RM70,000); and supplemental healthcare coverage (yes or no). Q1 represents the poorest 20% of the Malaysian population while Q5 represents the richest 20%. Supplemental healthcare coverage encompassed healthcare benefits for government employees, pensioner benefits, government-specific funds for healthcare, personal health insurance, and employer-sponsored benefits.

##### Health need factors

Self-rated health (good to excellent, very poor to fair); and presence of at least one non-communicable disease (NCD) (yes or no), assessed from the questions “Have you ever been told by a doctor or assistant medical officer that you have: (1) diabetes; (2) high blood pressure; (3) high cholesterol?” were used as proxy measures for health needs. Respondents who answered “yes” to any of the conditions, were coded as “yes” to the “presence of at least one NCD” during the analysis. Oral health is integral to general health and thus influenced by NCDs such as the bidirectional relationship between diabetes and periodontal disease. Hence, the presence of NCDs may prompt referral to seek oral healthcare which in turn affects the OHSB of individuals [35–37].

### Statistical analysis

Secondary data analysis was conducted using STATA version 14 (Stata Corp, College Station, TX, USA). To describe the predisposing, enabling, and health need characteristics of the respondents as well as their self-reported recent dental problems, stratified by location, descriptive statistics were conducted, and sample weights were applied. The prevalence of self-reported dental problems among Malaysian adults was compared for each characteristic, for both urban and rural areas, and *p-*value < 0.05 was considered a statistically significant difference. The sample weights used for estimates were created by multiplying the inverse sampling probability, the non-response adjustment factor, and the post-stratification adjustment by age, gender, and ethnicity.

The Chi-Square test was used to compare the characteristics of Malaysian adults from urban and rural areas. Using univariate and multivariable logistic regression analysis presented as crude odd ratios (CORs) and adjusted odd ratios (AORs) with 95% confidence intervals (CIs), characteristics of Malaysian adults who sought care from HCPs and those who self-medicated were predicted. In the univariate analysis, variables with a* p*-value of less than 0.25 were included in the multivariable regression analysis [38]. To analyse the variables that affected "sought healthcare" and "self-medication" using two models while controlling for all other variables, the multivariable analysis was carried out separately for urban and rural areas. Then, the AOR with a 95% confidence interval (CI) was calculated, with a *p*-value of less than 0.05 being deemed statistically significant.

To test for multicollinearity, the variance inflation factor (VIF) was used. A VIF of more than 10 indicates a potential problem with multicollinearity [39]. The `-lroc-` command was used to construct a Receiver Operating Characteristic (ROC) curve. The ROC curve, a well-established graphical metric for assessing model fit in logistic regression, effectively illustrates the balance between sensitivity and specificity. The Area Under the ROC Curve (AUC) was utilized to assess the goodness of fit of the models. An AUC of 0.9 to 1.0 was considered exceptional, 0.8 to 0.9 very good, 0.7 to 0.8 good, 0.6 to 0.7 sufficient, 0.5 to 0.6 poor, and less than 0.5 ineffective [40].

## Results

The analysis comprised a total of 10,134 respondents, representing about 18.2 million Malaysian adults aged 18 and above. The respondents comprised 76.3% urban and 23.7% rural population. Table 1 summarizes the predisposing, enabling, and health need factors of the respondents, stratified by location. Except for marital status and the existence of at least one NCD, there were substantial differences between urban and rural populations across all variables.

**Table 1:** Sociodemographic characteristics of respondents (n = 10,134)

Characteristics	n	Estimated population	Weighted %	95% CI		Location								*p*-value
						**Urban**				**Rural**				
						**n**	**Weighted %**	**95% CI**		**n**	**Weighted %**	**95% CI**		
				**LL**	**UL**			**LL**	**UL**			**LL**	**UL**	
**Overall**	10,134	18,262,522	100.0	-	-	6,071	76.3	74.5	78.0	4,063	23.7	22.0	25.5	-
***Predisposing factors***														
**Sex**														
Male	4,781	8,883,519	48.6	47.3	50.0	2,861	50.1	48.5	51.7	1,920	43.9	41.5	46.3	** < 0.001**
Female	5,353	9,379,003	51.4	50.0	52.7	3,210	49.9	48.3	51.5	2,143	56.1	53.7	58.5	
**Ethnicity**														
Malay^a^	6,995	10,435,997	57.1	52.8	61.4	3,833	52.8	47.5	58.0	3,162	71.2	65.6	76.2	** < 0.001**
Non-Malay	3,139	7,826,526	42.9	38.6	47.2	2,238	47.2	42.0	52.5	901	28.8	23.8	34.4	
**Age (years)**														
18–34	3,150	7,520,071	41.2	39.7	42.7	1,987	41.1	39.2	42.9	1,163	41.6	39.1	44.0	** < 0.001**
35–59	4,672	7,681,423	42.1	40.7	43.5	2,916	43.6	41.9	45.3	1,756	37.1	35.0	39.3	
60 +	2,312	3,061,029	16.8	15.5	18.1	1,168	15.3	13.9	16.9	1,144	21.3	19.0	23.8	
**Education level**														
No formal	516	686,101	3.8	3.3	4.3	215	2.7	2.2	3.4	301	7.1	6.1	8.2	** < 0.001**
Primary	2,130	3,202,730	17.5	16.3	18.9	972	14.8	13.4	16.4	1,158	26.3	24.0	28.8	
Secondary	4,927	9,265,577	50.7	49.0	52.5	2,981	51.2	49.1	53.3	1,946	49.2	46.7	51.7	
Tertiary	2,561	5,108,115	28.0	26.1	30.0	1,903	31.3	28.9	33.7	658	17.4	14.9	20.2	
**Marital status**														
Single	2,139	5,145,131	28.2	26.5	29.9	1,354	28.5	26.5	30.6	785	27.2	24.6	30.0	0.133
Married	6,866	11,540,085	63.2	61.4	65.0	4,085	63.3	61.1	65.5	2,781	62.7	59.8	65.5	
Not married^b^	1,129	1,577,306	8.6	7.8	9.5	632	8.2	7.8	9.5	497	10.1	8.9	11.5	
***Enabling factors***														
**Employment status**														
Government	1,124	1,445,188	7.9	7.0	9.0	768	8.0	6.9	9.3	356	7.6	6.1	9.5	** < 0.001**
Private	2,775	6,121,094	33.5	31.6	35.5	1,946	38.1	35.7	40.6	829	18.7	16.2	21.5	
Self-employed	1,931	3,418,402	18.7	17.4	20.1	935	16.7	15.2	18.4	996	25.1	22.6	27.8	
Unemployed	4,304	7,277,838	39.9	38.2	41.5	2,422	37.2	45.5	51.6	1,882	48.5	45.5	51.6	
**Income level**														
Q1 (RM0 – RM1,100)	2,206	3,811,908	20.9	19.3	22.5	1,039	17.1	15.4	18.9	1,167	33.1	29.7	36.7	** < 0.001**
Q2 (RM1,108 – RM2,100)	1,937	3,294,700	18.0	16.5	19.7	997	15.6	13.8	17.6	940	25.8	22.9	28.9	
Q3 (RM2,103 – RM3,400)	1,950	3,652,054	20.0	18.0	22.2	1,153	20.5	18.1	23.2	797	18.2	15.6	21.2	
Q4 (RM3,410 – RM5,900)	2,018	3,655,678	20.0	18.2	22.0	1,370	22.3	20.0	24.8	648	12.6	10.4	15.2	
Q5 (RM5,930 – RM70,000)	2,023	3,848,182	21.1	18.7	23.7	1,512	24.4	21.5	27.7	511	10.2	7.9	13.2	
**Supplemental healthcare coverage**														
Yes	5,395	10,062,046	55.1	52.9	57.3	3,722	60.5	57.8	63.1	1,673	37.8	34.3	41.3	** < 0.001**
No	4,739	8,200,476	44.9	42.7	47.1	2,349	39.5	36.9	42.2	2,390	62.2	58.7	65.7	
***Health need factors***														
**Self-rated health**														
Good to excellent	7,601	14,354,360	78.6	77.0	80.1	4,646	80.2	78.5	81.9	2,955	73.4	70.2	76.4	** < 0.001**
Fair	2,283	3,536,412	19.4	18.0	20.8	1,282	17.8	16.2	19.5	1,001	24.3	21.6	27.3	
Very poor to poor	250	371,750	2.0	1.7	2.4	143	2.0	1.6	2.4	107	2.3	1.7	3.0	
**Presence of at least one non-communicable diseases**														
Yes	3,021	4,468,542	24.5	23.2	25.8	1,740	24.2	22.6	25.8	1,281	25.4	23.7	27.3	0.296
No	7,113	13,793,980	75.5	74.2	76.8	4,331	75.8	74.2	77.4	2,782	74.6	72.7	76.3	

*n* count, *%* Percentage, *CI* Confidence Interval, *LL* Lower Limit, *UL* Upper Limit, *Q* Quintile

^a^Malay includes Orang Asli

^b^Not married includes divorced, separated or never married

Table 2 presents the prevalence of Malaysian adults who self-reported dental problems. The overall prevalence of Malaysian adults who self-reported dental problems was 5.5%. Of these, almost half (46.4%) sought healthcare, and slightly more than one-fifths (21.4%) self-medicated. The prevalence of Malaysian adults in the rural areas who self-reported dental problems (6.2%) was slightly higher than the urban adults (5.3%). There were significant differences in the prevalence of those who self-reported dental problems by different sociodemographic characteristics. Among the urban population, a higher prevalence of self-reported dental problems was seen among middle aged adults (35–59 years), married individuals, respondents with NCD, those who self-rated their health as very poor to poor, and those with supplemental healthcare coverage. Among the rural population, a higher prevalence of self-reported dental problems was seen among non-Malays, middle aged adults (35–59 years), respondents with NCD, and those who self-rated their health as very poor to poor.

**Table 2:** Prevalence of Malaysian adults who self-reported dental problems, stratified by location

Characteristics	Total (*n* = 10,134)				Location									
					**Urban (*n* = 6,071)**					**Rural (*n* = 4,063)**				
	**n**	**Weighted %**	**95% CI**		**n**	**Weighted** **%**	**95% CI**		***p*-value**	**n**	**Weighted %**	**95% CI**		***p*-value**
			**LL**	**UL**			**LL**	**UL**				**LL**	**UL**	
**Overall**	603	5.5	4.8	6.2	378	5.3	4.5	6.2	0.192	225	6.2	5.2	7.3	0.192
***Predisposing factors***														
**Sex**														
Male	260	5.2	4.3	6.3	169	5.0	4.0	6.2	0.409	91	6.2	4.7	8.2	0.984
Female	343	5.7	4.9	6.7	209	5.6	4.6	6.8		134	6.2	4.7	8.0	
**Ethnicity**														
Malay^a^	424	5.3	4.6	6.1	263	5.2	4.3	6.2	0.898	161	5.4	4.3	6.7	**0.019**
Non-Malay	179	5.8	4.6	7.2	161	5.3	4.1	6.9		64	8.2	6.2	10.7	
**Age (years)**														
18–34	156	4.2	3.3	5.2	98	3.9	2.9	5.1	**0.004**	58	5.2	3.7	7.2	**0.030**
35–59	323	7.0	5.8	8.4	209	6.7	5.3	8.4		114	8.1	6.3	10.2	
60 +	124	5.0	3.9	6.3	71	5.0	3.6	6.9		53	4.9	3.5	6.8	
**Education level**														
No formal	32	5.6	3.5	8.7	13	4.9	2.3	10.0	0.937	19	6.4	3.6	11.1	0.975
Primary	132	5.8	4.5	7.6	60	5.7	3.9	8.3		72	6.1	4.4	8.2	
Secondary	284	5.6	4.6	6.6	179	5.3	4.2	6.6		105	6.3	5.0	8.0	
Tertiary	155	5.1	4.0	6.5	126	5.0	3.8	6.6		29	5.8	3.4	9.6	
**Marital status**														
Single	94	3.6	2.6	4.8	57	2.9	1.9	4.3	** < 0.001**	37	5.8	3.7	8.8	0.494
Married	435	6.4	5.5	7.5	278	6.3	5.2	7.7		157	6.6	5.3	8.2	
Not married^b^	74	5.0	3.7	6.8	43	5.1	3.4	7.5		31	4.7	3.2	7.0	
***Enabling factors***														
**Employment status**														
Government	74	6.2	4.3	8.9	62	7.0	4.6	10.3	0.578	12	3.8	1.9	7.4	0.254
Private	150	5.3	4.1	7.0	104	5.2	3.8	7.1		46	6.0	4.1	8.8	
Self-employed	124	5.8	4.6	7.3	60	4.9	3.5	6.7		64	7.8	5.7	10.5	
Unemployed	255	5.3	4.4	6.3	152	5.1	4.1	6.4		103	5.8	4.3	7.7	
**Income level:**														
Q1 (RM0 – RM1,100)	141	5.8	4.6	7.4	66	5.3	3.6	7.5	0.276	75	6.8	5.3	8.7	0.154
Q2 (RM1,108 – RM2,100)	101	4.5	3.4	5.9	51	3.6	2.5	5.2		50	6.2	4.1	9.3	
Q3 (RM2,103 – RM3,400)	122	6.5	5.1	8.4	75	6.4	4.7	8.7		47	7.1	4.6	10.7	
Q4 (RM3,410 – RM5,900)	116	5.1	3.9	6.6	79	4.9	3.6	6.6		37	6.4	4.1	9.9	
Q5 (RM5,930 – RM70,000)	123	5.3	3.8	7.3	107	5.7	4.1	8.0		16	2.1	1.0	4.5	
**Supplemental healthcare coverage**														
Yes	342	5.9	4.9	7.0	261	6.0	4.9	7.3	**0.015**	81	5.0	3.8	6.6	0.106
No	261	5.0	4.2	6.0	117	4.1	3.2	5.3		144	6.9	5.4	8.6	
***Health need factors***														
**Self-rated health**														
Good to excellent	370	4.5	3.8	5.3	233	4.3	3.5	5.3	** < 0.001**	137	5.3	4.1	6.7	**0.024**
Fair	206	8.9	7.4	10.7	128	9.2	7.3	11.5		78	8.3	6.1	11.1	
Very poor to poor	27	10.1	6.1	16.1	17	9.2	5.0	16.2		10	12.6	5.3	27.1	
**Presence of at least one non-communicable diseases**														
Yes	232	7.6	6.3	9.1	141	7.2	5.7	9.1	**0.003**	91	8.7	6.6	11.4	**0.002**
No	371	4.8	4.1	5.6	237	4.6	3.8	5.7		134	5.3	4.3	6.5	
***Health seeking behaviour***														
**Sought healthcare**														
Yes	299	46.4	40.7	52.1	184	47.6	40.8	54.6	0.436	115	42.9	33.7	52.7	0.436
No	304	53.6	47.9	59.3	194	52.4	45.4	59.2		110	57.1	47.3	66.3	
**Self-medicated**														
Yes	475	21.4	17.0	26.6	291	22.9	17.4	29.5	0.242	184	17.3	11.5	25.3	0.242
No	128	78.6	73.4	83.0	87	77.1	70.5	82.6		41	82.7	74.7	88.5	

*n* count, % Percentage, *CI* Confidence Interval, *LL* Lower Limit, *UL* Upper Limit, *Q* Quintile

^a^Malay includes Orang Asli

^b^Not married includes divorced, separated or never married

Table 3 displays the results of the logistic regression model for OHSB of Malaysian adults who self-reported dental problems, stratified by location. Model I and II assessed the factors associated with seeking healthcare among Malaysian adults who self-reported dental problems in urban and rural areas, respectively. The multivariable logistic regression among urban population revealed that ethnicity was associated with seeking healthcare. Among urban dwellers, Malays were more likely than non-Malays to seek treatment from HCPs (AOR = 2.11, 95% CI: 1.10–4.03). Among the rural population, income was associated with seeking healthcare. The richest 20% of rural dwellers were more likely than the poorest 20% to seek treatment from HCPs (AOR = 14.00, 95% CI: 2.89 – 67.56).

**Table 3:** Logistic regression model for oral health-seeking behaviour of Malaysian adults who self-reported dental problems, stratified by location

Factors	Sought treatment from HCP								Self-medicated							
	**Model I – Urban**				**Model II—Rural**				**Model III—Urban**				**Model IV—Rural**			
	**Crude Odds Ratio, OR (95% CI)**	***p*-value**	**Adjusted Odds Ratio, OR (95% CI)**	***p*-value**	**Crude Odds Ratio, OR (95% CI)**	***p*-value**	**Adjusted Odds Ratio, OR (95% CI)**	***p*-value**	**Crude Odds Ratio, OR (95% CI)**	***p*-value**	**Adjusted Odds Ratio, OR (95% CI)**	***p*-value**	**Crude Odds Ratio, OR (95% CI)**	***p*-value**	**Adjusted Odds Ratio, OR (95% CI)**	***p*-value**
**Sex**																
Male	1.00 (ref)				1.00 (ref)				1.00 (ref)				1.00			
Female	1.24 (0.67—2.27)	0.481			1.50 (0.70 – 3.19)	0.290			0.95 (0.48—1.85)	0.882			0.59 (0.19—1.78)	0.348		
**Ethnicity**																
Malay^a^	**2.03 (1.07 – 3.83)**	**0.029**	**2.11 (1.10 – 4.03)**	**0.023**	0.94 (0.43 – 2.01)	0.863			**2.55 (1.24 – 5.24)**	**0.011**	**2.62 (1.26 – 5.45)**	**0.010**	0.90 (0.34 – 2.38)	0.836		
Non-Malay	1.00 (ref)		1.00 (ref)		1.00 (ref))				1.00 (ref)		1.00 (ref)		1.00 (ref)			
**Age (years)**																
18–34	1.00 (ref)		1.00 (ref)		1.00 (ref)				1.00 (ref)				1.00			
35–59	1.49 (0.79—2.81)	0.212	1.33 (0.73 – 2.43)	0.352	0.60 (0.23—1.55)	0.293			0.97 (0.46 – 2.04)	0.941			1.06 (0.33—3.42)	0.911		
60 +	1.88 (0.84—4.20)	0.120	1.42 (0.58 – 3.50)	0.435	0.70 (0.20 – 2.38)	0.572			0.85 (0.30 – 2.39)	0.760			0.92 (0.25—3.33)	0.901		
**Education level**																
No formal	1.00 (ref)				1.00 (ref)				1.00 (ref)		1.00 (ref)		1.00 (ref)		1.00 (ref)	
Primary	1.66 (2.81—9.88)	0.574			1.08 (0.30 – 3.85)	0.909			0.25 (0.04 – 1.55)	0.137	0.32 (0.05 – 1.85)	0.199	0.43 (0.10 – 1.93)	0.271	0.36 (0.08 – 1.58)	0.174
Secondary	2.03 (0.38 – 11.00)	0.406			0.77 (0.22 – 2.61)	0.667			0.81 (0.14 – 4.56)	0.808	0.67 (0.13 – 3.38)	0.625	0.37 (0.07 – 1.99)	0.244	0.25 (0.04 – 1.47)	0.124
Tertiary	1.97 (0.34—11.26)	0.445			2.01 (0.45 – 9.13)	0.361			1.02 (0.18 – 5.98)	0.980	0.70 (0.13 – 3.83)	0.678	**0.01 (0.00—0.07)**	** < 0.001**	**0.01 (0.00 – 0.03)**	** < 0.001**
**Marital status**																
Single	0.54 (0.24—1.22)	0.142	0.70 (0.32—1.50)	0.352	2.15 (0.79 – 5.82)	0.129	1.86 (0.67 – 5.16)	0.234	1.90 (0.81 – 4.41)	0.135	2.01 (0.86 – 4.72)	0.108	0.60 (0.16—2.13)	0.428		
Married	1.00 (ref)		1.00 (ref)		1.00 (ref)		1.00 (ref)		1.00 (ref)		1.00 (ref)		1.00 (ref)			
Not married^b^	1.68 (0.70—4.03)	0.242	1.43 (0.56—3.66)	0.458	1.12 (0.38 – 3.31)	0.831	1.58 (0.38 – 6.55)	0.526	0.59 (0.20 – 1.70)	0.334	0.90 (0.25 – 3.29)	0.875	1.53 (0.44—5.29)	0.495		
**Employment status**																
Public employee	1.54 (0.69 – 3.41)	0.291			0.73 (0.15 – 3.52)	0.692	0.41 (0.08 – 2.10)	0.281	1.58 (0.62 – 4.02)	0.339			0.78 (0.01 – 7.68)	0.827		
Private employee	0.78 (0.41 – 1.50)	0.452			1.12 (0.42 – 2.97)	0.818	0.95 (0.35 – 2.55)	0.912	0.99 (0.47 – 2.13)	0.989			1.37 (0.36 -5.29)	0.644		
Self-employed	1.18 (0.59 – 2.37)	0.633			0.48 (0.20 – 1.19)	0.112	0.44 (0.19 – 1.05)	0.064	0.56 (0.20 – 1.55)	0.263			1.70 (0.58 – 4.99)	0.329		
Unemployed	1.00 (ref)				1.00 (ref)		1.00 (ref)		1.00 (ref)				1.00 (ref)			
**Income**																
Q1 (RM0 – RM1,100)	1.00 (ref)				1.00 (ref)		1.00 (ref)		1.00 (ref)		1.00 (ref)		1.00 (ref)		1.00 (ref)	
Q2 (RM1,108 – RM2,100)	1.17 (0.40 – 3.37)	0.777			3.46 (1.37 – 8.73)	0.009	**3.64 (1.38 – 9.58)**	**0.009**	0.85 (0.23 –3.13)	0.808	0.71 (0.17 – 3.00)	0.641	1.13 (0.29 – 4.46)	0.860	2.02 (0.54 – 7.52)	0.292
Q3 (RM2,103 – RM3,400)	1.65 (0.61 –4.50)	0.326			1.06 (0.40 – 2.78)	0.903	1.53 (0.58 – 4.06)	0.388	1.81 (0.59 – 5.56)	0.296	1.43 (0.42 – 4.85)	0.569	1.85 (0.48 – 7.08)	0.366	3.43 (0.84 – 13.96)	0.085
Q4 (RM3,410 – RM5,900)	0.90 (0.39—2.10)	0.809			3.76 (1.35 – 10.49)	0.012	**5.64 (1.76 – 18.11)**	**0.004**	0.81 (0.27 – 2.45)	0.710	0.68 (0.19 – 2.46)	0.560	0.18 (0.02—1.25)	0.082	0.33 (0.04 – 2.60)	0.293
Q5 (RM5,930 – RM70,000)	1.41 (0.57 – 3.49)	0.460			8.03 (1.66 – 38.78)	0.010	**14.0 (2.89 – 67.56)**	**0.001**	2.25 (0.79 – 6.43)	0.129	1.47 (0.47 – 4.59)	0.504	1.22 (0.20 – 7.37)	0.825	6.86 (1.00 – 46.90)	0.05
**Supplemental financial coverage**																
Yes	0.94 (0.53—1.68)	0.858			0.75 (0.33—1.70)	0.498			1.18 (0.61 – 2.26)	0.616			0.64 (0.20—2.05)	0.455		
No	1.00 (ref)				1.00 (ref)				1.00 (ref)				1.00 (ref)			
**Self-rated health**																
Good to excellent	1.00 (ref)		1.00 (ref)		1.00 (ref)		1.00 (ref)		1.00 (ref)				1.00 (ref)			
Very poor to fair	1.51 (0.83—2.73)	0.174	1.42 (0.74—2.70)	0.289	1.81 (0.80 – 4.13)	0.156	1.94 (0.84 – 4.51)	0.118	0.69 (0.36 – 1.31)	0.251			1.17 (0.43—3.15)	0.757		
**Presence of at least one non-communicable diseases**																
Yes	1.26 (0.69—2.31)	0.436			0.80 (0.36 – 1.76)	0.589			0.80 (0.41—1.54)	0.506			1.37 (0.52—3.55)	0.515		
No	1.00 (ref)				1.00 (ref)				1.00 (ref)				1.00 (ref)			

Model I assessed the factors associated with sought healthcare treatment among self-reported dental problems adults in urban location; Model II assessed the factors associated with sought healthcare treatment among self-reported dental problems adults in rural location; Model III assessed the factors associated with self-medicated among self-reported dental problems adults in urban location; Model IV assessed the factors associated with self-medicated among self-reported dental problems adults in rural location. Total n for Model I and III: 6,071; Model II and IV: 4,063. Area under ROC curve for Model I: Urban = 0.5572; Model II: Rural = 0.6777; Model III: Urban = 0.6160; Model IV: Rural = 0.6494. *OR* Crude Odd Ratios *CI* Confidence Interval, *Q* Quintile

^a^Malay includes Orang Asli

^b^Not married includes divorced, separated or never married

Model III and IV assessed the factors associated with self-medication among Malaysian adults who self-reported dental problems in urban and rural areas, respectively. The regression revealed that ethnicity was associated with self-medication among urban dwellers, where Malays (AOR = 2.62, 95% CI: 1.26 – 5.45) were more likely than non-Malays to self-medicate. Among the rural population, education level was associated with self-medication. Rural dwellers with tertiary education (AOR = 0.01, 95% CI: 0.00 – 0.03) were less likely than rural dwellers with no formal education to self-medicate.

All the models except Model I had an AUC of more than 0.6. Model I had an AUC between 0.5 and 0.6, which is lower than the others. However, the sensitivity and specificity of Model I were 73.7% and 33.2%, respectively. In this study, a sensitivity of 73.7% is adequate to correctly identify the proportion of respondents who sought healthcare from a healthcare practitioner. In addition, the specificity of Model I, which predicts the proportion of respondents who did not seek healthcare from a HCP, is of less concern in our study, since the other models including those predicting self-medication have an AUC of more than 0.6 [41]. Therefore, the models were considered fit. Multicollinearity is the occurrence of high intercorrelations among two or more independent variables in a multiple regression model. It is important to ensure multicollinearity is low in logistic regression analysis. In the present study, multicollinearity analysis using Variance Inflation Factors (VIF) showed that multicollinearity was unlikely as the VIFs were less than 5 (ranging from 1.02 to 1.97).

## Discussion

This study aimed to determine the prevalence and characteristics of Malaysian adults who self-reported dental problems based on their urban–rural location, as well as the factors associated with their OHSBs. All variables, excluding marital status and the presence of any NCD, were substantially different between the urban and rural populations.

The overall prevalence of Malaysian adults who self-reported dental problems was low (5.5%) and was slightly higher in the rural population than the urban population. Studies have found that illnesses were more prevalent among rural population [21, 22]. The present study used self-reported dental problems rather than objective measures of dental problems and untreated dental needs. However, previous research demonstrated the adequacy of the subjective measure in predicting objective dental needs [42] and the links between the measure of self-reported dental problems and psychosocial factors [43]. While self-reported dental problems are a limited measure of oral health, it is a significant predictor of having unmet dental treatment needs [42]. For example, data from the National Oral Health Survey of Adults (NOHSA) 2010 found more than half of the adults perceived that their oral health was excellent/good [44] but a national study conducted in 2019 found that 29.9% of the population in Malaysia perceived the need to seek care for their recent oral health problems but did not do so [20], indicating unmet dental treatment needs.

Medication is crucial in managing dental issues by alleviating pain, controlling infections, and promoting oral health [45]. This includes pain relievers, antibiotics for bacterial infections, antiseptics, and topical treatments. However, the practice of self-medication, particularly with antibiotics, poses significant concerns. Self-medication can lead to antibiotic misuse, where incomplete courses, inadequate diagnoses, and overuse of broad-spectrum antibiotics contribute to antibiotic resistance [46]. This relationship underscores the importance of seeking professional guidance from dentists to ensure effective and responsible treatment for dental problems, safeguarding against potential complications and the global threat of antibiotic resistance.

Ethnicity was associated with seeking healthcare and self-medication among urban dwellers where non-Malays were less likely than Malays to seek treatment from HCPs nor self-medicate. This finding contradicts previous research in Sarawak where urban dwellers and the Chinese were more likely to seek dental care [47]. Another study among Malaysian elderly revealed that while Malays and Indians were more frequent users of emergency dental services, Chinese were found to be high users of rehabilitative dental treatments [48]. However, both these studies involved a small population compared to the large nationwide population in the current study. Hence, further research is required to explore the reasons for the association between ethnicity and OHSB among urban dwellers in Malaysia as depicted in this study.

The accessibility of Malaysia's public sector is bolstered by its nominal fees for utilization [49], a factor that actively promotes its use. This is further underscored by exemptions from these fees for civil servants, with Malays constituting the majority among them [50]. Notably, the Urban and Housing Census of 2000 revealed that Malays constituted the largest proportion of the urban population at 43.9%, followed by the Chinese at 33.9%, and Indians at 9.3% [51]. Among Malays, there exists an urban impoverished demographic [52] that might resort to self-medication as a means to alleviate discomfort, as pressing essentials such as food security take precedence over their oral well-being. Factors such as limited health literacy, unfavorable attitudes towards oral hygiene, and a lack of perceived importance in oral disease prevention also contribute to this trend [38]. This information is integral for understanding the propensity of Malays, in comparison to non-Malays, to seek healthcare services. To improve healthcare accessibility and utilization among urban impoverished Malays, the Malaysian government can implement targeted programs, such as subsidies and special clinics, that address their specific challenges without compromising essential needs like food security. Community outreach efforts can directly bring healthcare services to these underserved areas, overcoming geographical and financial barriers. Recognizing the importance of civil servants, particularly Malays, in healthcare utilization, the government can offer incentives that encourage both seeking healthcare and promoting awareness within their communities. To ensure effectiveness, healthcare initiatives should also be culturally sensitive and respect local values, especially when addressing topics like oral hygiene and healthcare-seeking behavior.

Among the rural population, income level was associated with seeking healthcare while education level was associated with self-medication. The poorest 20% of rural dwellers were less likely than the richest 20% to seek treatment from HCPs. Predisposing variables indicate inequalities between urban and rural areas. Though healthcare in the public sector is heavily subsidised by the Malaysian government, patient waiting time at public dental clinics are often lengthy due to congestion. Additionally, compared to metropolitan people, rural regions have fewer hospital beds, doctors, nurses, and specialists per capita [53]. Moreover, distance to health facilities could also be far in rural areas and those from lower socioeconomic status may not have the transportation or may not be able to afford the cost of transportation to the facilities. Affordability of health and dental services in private facilities could be another significant reason for not seeking HCPs among low socioeconomic status residents from rural areas. This is in line with a study of older persons in 14 European countries that indicated those with lower incomes were less likely to seek dental care [54]. To enhance healthcare utilization, especially among rural residents and those with lower socioeconomic status, the Malaysian government could adopt the following measures: improve accessibility by addressing congestion at public dental clinics through increased infrastructure and technology investment; expand healthcare infrastructure, incentivize HCPs to serve underserved areas, and establish new facilities in rural regions; tackle transportation challenges by offering affordable transport options to health facilities; enhance affordability with targeted subsidies for health and dental services; establish strategically located rural health clinics offering comprehensive care; launch health education campaigns to raise awareness and dispel misconceptions; deploy community health workers to offer guidance and assistance; provide financial support like subsidies to lower socioeconomic groups; collaborate with private healthcare for accessible services through partnerships or negotiated pricing; and integrate telehealth services to offer remote consultations, benefiting those facing transportation issues.

It is imperative to address education, which the World Health Organization considers as one of the key socioeconomic determinants of health, in order to improve health and diminish persistent health disparities [55]. In our study, individuals who had higher education in rural areas were less likely to self-medicate than those with lower education. A possible explanation could be that those with tertiary education may have some awareness about the risks of drug misuse and may have better monetary and logistic resources to seek treatment from healthcare institutions in rural areas. According to a Saudi Arabian study, education level has an impact on the use of self-medication [56]. While it has been established that over-the-counter medicines can be taken safely and effectively without a doctor's supervision, using them carelessly could have unintended consequences [57]. The likelihood that low health literacy among the less educated coupled with easy access to medicine, may have disastrous repercussions, which motivates the need to improve health literacy, particularly the harmful implications of self-medication to one's wellbeing [58]. According to Malaysia's national health plan, "Agenda Nasional Malaysia Sihat" (ANMS), campaigns like "Know Your Medicines" could promote the value of medicine knowledge in order to raise public awareness and strengthen individual health [59].

Self-medication reduces the need for professional healthcare, but it is associated with a number of potential hazards [60–63]. This issue highlights the importance of HCPs in promoting reasonable pharmaceutical consumption and providing information on potential side effects to promote informed and responsible self-medication [60]. In our study, we classified community pharmacists as healthcare practitioners (HCPs). Therefore, when individuals sought guidance from a community pharmacist for medication, it signified that they had sought care from HCPs. It was important to distinguish that, in our study, self-medication involved using medication without input from HCPs. It was worth noting that seeking guidance from a community pharmacist should not be confused with self-medication. Self-medication specifically referred to individuals independently deciding to take medication without the guidance or prescription of a healthcare practitioner [64]. It was crucial to recognize that self-medication could be risky and might have led to adverse effects if not executed properly. In contrast, seeking guidance from a pharmacist constituted seeking HCP assistance, even if the pharmacist did not prescribe medication. Pharmacists were skilled HCPs capable of offering information and advice on over-the-counter medications, potential drug interactions, and appropriate medication usage. Seeking guidance from a pharmacist represented a pivotal facet of responsible self-care and contributed to ensuring the secure and effective use of medications. Pharmacists played a critical role in promoting patient education and upholding medication safety [65]. Additionally, to aid in the understanding of oral disease processes and beneficial oral health practices, public health awareness campaigns can be conducted as a component of larger public health initiatives. A targeted approach in spreading awareness to the Malaysian population may be adopted based on the findings of the present study. For instance, community pharmacists can be used to raise knowledge about medication safety and oral health among urban residents. Given that Malaysians with lower income in the rural areas are more inclined to self-medicate, pharmacists in public hospitals have a stronger role in educating the populace there about medication safety and oral health where community pharmacies are fewer [66] and the use of public health facilities are more prevalent [15]. Oral health personnel may collaborate with pharmacists in the hospital to sensitize them about study findings which show preponderance for self-medication and its associated factors among Malaysians. This can be done during continuing medical education (CME) sessions organized by oral health personnel or the respective hospitals.

The present study's strength is in its comparisons of Malaysian residents who live in urban and rural areas. This is one of the first investigations assessing differences between urban and rural people in oral health seeking behaviour in Malaysia. Furthermore, the study's sample size was sizable, consisting of 10,134 adults from both urban and rural locations. This research has some weaknesses despite its advantages. No causal link could be established between healthcare seeking behavior and associated characteristics due to the cross-sectional nature of this study. The data was only taken at one point in time, making it impossible to measure seasonal change. In addition, the NHMS 2019 did not include clinical examination of the respondents. Hence, it was not possible to correlate self-reported responses to clinical findings. Another limitation is that the type of dental problems was not asked specifically and hence it was not possible to pin-point the type of dental problem faced by each respondent. The type of questions that required “yes” or “no” answers also limited the respondents’ choices, wherein a “no” could have meant that they did not have any dental problems or something else that was not explicitly stated in the questionnaire. Finally, there is a chance of recall bias because our investigation relied on self-reported information about past events.

## Conclusion

The results of this cross-sectional study revealed that ethnicity, income level, and education level, were factors linked with OHSBs among Malaysian individuals who self-reported dental problems from both urban and rural settings. Although Malaysia has achieved Universal Health Coverage (UHC), this study reveals oral healthcare inequalities driven by variations in OHSBs between urban and rural areas, highlighting the need for further improvement in the provision of oral healthcare services. Future policies should adopt more focused strategies that concentrate on the vulnerable and rural populations, particularly with regards to accessibility of oral healthcare services and their health knowledge and literacy on how to seek the best oral healthcare. Oral health maintenance ought to be ingrained in society as a way of life. Social media and the mainstream media are also significant forces in educating the public about oral health issues. Further insights on the Malaysian population's OHSB may be gained through additional in-depth studies on aspects like perceived service quality.

## Data Availability

To protect the privacy of the participants, the dataset that supports the findings of this article is not publicly available. The data may be requested from the corresponding author on reasonable request upon permission from the Director General of Health, Malaysia.

## Abbreviations

AOR: Adjusted Odds Ratio
AUC: Area Under the Curve
CI: Confidence Interval
CME: Continuing Medical Education
DOSM: Department of Statistics, Malaysia
EBs: Enumeration Blocks
HCP: Healthcare Practitioner
LQs: Living Quarters
MOH: Ministry of Health
MREC: Medical Research and Ethics Committee
NCD: Non-communicable disease
NHMS: National Health and Morbidity Survey
NIH: National Institutes of Health, Malaysia
NMRR: National Medical Research Register
NOHSA: National Oral Health Survey of Adults
OHSB: Oral Healthcare Seeking Behavior
ROC: Receiver Operating Characteristic
SD: Secure Digital
UHC: Universal Health Coverage
VIF: Variance Inflation Factor
